# CLASP promotes stable tethering of endoplasmic microtubules to the cell cortex to maintain cytoplasmic stability in *Arabidopsis* meristematic cells

**DOI:** 10.1371/journal.pone.0198521

**Published:** 2018-06-12

**Authors:** P. Yen Le, Chris Ambrose

**Affiliations:** Department of Biology, The University of Saskatchewan, College of Arts and Science, Saskatoon, SK Canada; Iowa State University, UNITED STATES

## Abstract

Following cytokinesis in plants, Endoplasmic MTs (EMTs) assemble on the nuclear surface, forming a radial network that extends out to the cell cortex, where they attach and incorporate into the cortical microtubule (CMT) array. We found that in these post-cytokinetic cells, the MT-associated protein CLASP is enriched at sites of EMT-cortex attachment, and is required for stable EMT tethering and growth into the cell cortex. Loss of EMT-cortex anchoring in *clasp-1* mutants results in destabilized EMT arrays, and is accompanied by enhanced mobility of the cytoplasm, premature vacuolation, and precocious entry into cell elongation phase. Thus, EMTs appear to maintain cells in a meristematic state by providing a structural scaffold that stabilizes the cytoplasm to counteract actomyosin-based cytoplasmic streaming forces, thereby preventing premature establishment of a central vacuole and rapid cell elongation.

## Introduction

In plants, the microtubule (MT) cytoskeleton is a central player in a multitude of developmental and environmental roles ranging from cell division, expansion, hormonal signalling, tropisms, to biotic and abiotic stress. During these diverse processes, the primary role of MTs is to modulate cell wall formation and structure. In particular, MTs influence the structure and orientation of cellulose microfibrils within the cell wall, which then determines cell shape by fostering non-uniform cell enlargement in response to uniform intracellular turgor pressure. During interphase, MTs are categorized into cortical microtubules (CMTs), which line the cell membrane, and endoplasmic microtubules (EMTs), which reside within the cytoplasm. CMTs assist in guiding the linear movement of membrane-associated cellulose synthase complexes as they extrude cellulose microfibrils into the cell wall [1–4]. CMTs take on a variety of cell-specific configurations in order to precisely transmit intracellular information to the extracellular matrix. Generally speaking, alignment of CMTs into parallel arrays drives cell expansion perpendicularly to orientation of the CMTs, while mixed/net-like configurations promote isotropic expansion.

In contrast to CMTs, which are found in essentially all vacuolated cell types, EMTs are more specialized, being abundant in cytoplasmically dense cells, such as meristematic cells and tip-growing cells, such as root hairs and pollen tubes. While EMTs generally have no connections to the cell cortex in tip-growing cells, in meristematic cells, they form a radial network connecting the nucleus and cell cortex [5,6]. This radial EMT configuration is thought to influence cell expansion indirectly, by randomizing the CMT array. Specifically, by growing into the cell cortex in multiple directions, EMTs foster mixed CMT orientations, which limit cell elongation and promote isotropic cell expansion [7,8]. EMT abundance is highest following cytokinesis, and then gradually dissipates as cells initiate elongation, which is accompanied by the gradual development of a central vacuole, vigorous cytoplasmic streaming, and establishment of a transverse CMT array [7–12]. EMTs are also abundant in pre-mitotic cells, where they connect the nucleus to the preprophase MT band to assist in the normal formation of the mitotic spindle [13–17].

Although the continuity of the EMT-CMT systems is important to plant cell morphogenesis, the mechanisms underlying their relationship and how EMTs attach to the cortex is not well understood. Despite the large number of mutants in MT-associated Proteins (MAPs) that show defects in CMT array function, EMT phenotypes have not been widely documented. Mutations in the MT-severing protein KATANIN cause delayed disappearance of EMTs in meristematic cells and subsequent failure of CMTs to reorient from mixed to transverse, resulting in radially swollen root tips due to isotropic cell expansion [18,19]. However, given that *katanin* mutants also show highly disorganized CMT arrays in cell types that lack EMTs, it is difficult to tease out any specific contributions of EMTs to normal cell expansion.

CLASP (CLIP-Associating Protein) is an evolutionary conserved MAP belonging to the DIS/TOG family, and plays diverse roles in animals, fungi, and plants [20–25]. Arabidopsis contains a single copy of the *CLASP* gene, which has been implicated in a wide array of functions during cell division, expansion and differentiation [20,26–30]. Consistent with this, *clasp-1* null mutants have a dwarf phenotype, smaller apical meristems, and show generalized defects in cell division and expansion [20,30,31].

In animals, CLASPs are often enriched at growing MT plus-ends, where they locally stabilize MTs in order to facilitate attachment to intracellular structures such as the cell cortex and chromosomal kinetochores [23,32–35]. In contrast, Arabidopsis CLASP shows only a mild enrichment at MT plus-ends, instead distributing along the sidewall lattice of CMTs in a punctate manner in enlarging/mature cells, where it functions to maintain lateral attachment of CMTs with the plasma membrane [20,26,30]. In recently divided (i.e. post-cytokinetic) meristem cells, CLASP becomes heavily enriched at newly formed cell edges, where it modulates CMT array organization by facilitating CMT growth around sharp cell edges [36]. Passage of CMTs around cell edges promotes the formation of mixed/longitudinal CMT organization in division zone cells, which by interrupting formation of transverse CMTs, may be important in preventing premature cell elongation and exit from the meristem. This inability to generate mixed CMT arrays has been proposed as an explanation for the reduced meristem size in *clasp-1* mutants [36].

In the current study, we show a novel role for CLASP that reveals a new function for EMTs in plants.

## Methods

### Plant materials and growth conditions

Seeds were sterilized by 70% EtOH and put at 4°C in dark for 2 days for stratification. Plants were grown in continuous light conditions on vertical agar plates containing ½ MS, 0.7% Phytagel, and 1.0% Sucrose medium. Meristem cell zone in root tips were imaged at 5–7 days.

Wild type and *clasp-1* mutant *Arabidopsis thaliana* Columbia ecotype plants expressing CLASP::GFP-CLASP/UBQ::RFP-TUB6 were used for visualizing CLASPs and microtubules [36] and 35S::GFP-ɣTIP for visualizing vacuoles [37].

### Molecular biology and construct design

For UBQ::GFP-MBD, GFP-MBD fragment was amplified from existing *Arabidopsis thaliana* (Col-0) plants expressing 35S::GFP-MBD, and cloned into the SalI/SacI sites of a modified pCAMBIA1300 vector containing UBQ promoter [36]. Primers were fwd 5’ -cctaggatgagtaaaggagaagaacttttcactgg- 3’, and rev 5’ -tgtttgaacgatctgcagccg- 3’.

For CLASP::RFP-CLASP, TAGRFP [38] was generated via gene synthesis (Invitrogen DNA Strings) and cloned into a modified pCAMBIA2300 vector containing pCLASP::GFP-CLASP [36] to replace the position of GFP gene at the SalI/BamHI sites.

All constructs were transferred into *Agrobacterium tumefaciens* strain GV3101 and selected on LB medium containing 12.5 μg mL^-1^ tetracycline, 25 μg mL^-1^ gentamycin, and 50 μg mL^-1^kanamycin (Sigma-Aldrich) *A*. *tumefaciens* containing the constructs were transformed into plants by the floral dip method [39].

### Drug treatment and staining

Plants were grown vertically on MS media as described above. For treatment, roots were cutoff at the basal region near the junction with hypocotyl, and placed in coverslip chamber (Lab-Tek; Nunc) containing 50 μM Oryzalin in (0.5% DMSO) or 0.5% DMSO for mock treatments. Treatments were typically 30–60 min and are indicated in the figure legends.

For staining, after cut and placed in staining solution (100μM BCECF or10μM Propidium Iodide for 10 minutes, and 5μM Synaptored C2 (FM4-64;VWR International) for 2 hours the root tips were covered by solidified 1% agarose and imaged right after that.

### Immunofluorescence

*Arabidopsis* seedlings grown on vertical agar plates were fixed for 40 min in solution containing 4% formaldehyde (EMS; https://www.emsdiasum.com/microscopy/) and 0.1% glutaraldehyde (EMS; https://www.emsdiasum.com/microscopy/). Following fixation, plants were rinsed in PME (5mM PIPES + 2mM EGTA + 2mM MgSO_4_); and digested with cell-wall-degrading enzymes (0.05% cellulose, 0.1% pectolyase), with 0.2% Triton X-100 for 30 min. Specimens were then treated with 3% bovine serum albumin in PBS buffer for 1 h, rinsed, and incubated in DM1A α-tubulin antibody (1:1000 dilution; ThermoFisher Scientific) at 4°C overnight. Specimens were rinsed and incubated in Alexa Fluor® 488 goat anti-mouse IgG (H+L; 1:200 dilution) for 4 h. Specimens were rinsed and mounted in 25% Citifluour AF1 anti-fade agent.

### Tissue preparation and microscopy

For live cell imaging, roots were cut 0.5 cm above root tips, mounted in Nunc chambers (Lab-Tek) under Perfluoroperhydrophenanthrene (PP11) [40], and covered by 2~3-mm-thick 1% agarose. Images were obtained via point-scan confocal microscopy (Zeiss Meta 510 with Zeiss Axiovert 200M inverted microscope, 63X water immersion), or Zeiss 880 Airyscan with 63x water immersion for S2 Fig, S5 Movie and S6 Movie.

### Image analysis

Images were processed with Image J software (http://rsb.info.nih.gov/ij/) and 3DMOD software (http://bio3d.colorado.edu/imod/doc/3dmodguide.html). The ClearVolume plugin (https://imagej.net/ClearVolume) [41] from Image J was used for 3D renderings in S2 Fig and S5 Movie. Figures were assembled using Corel Draw software (www.Corel.com; Corel System. Ottawa, ON, Canada), and Inkscape vector graphics software (www.inkscape.org). Statistical analysis was performed using Microsoft Excel (Microsoft, Edmond, WA).

## Results

### CLASP localizes to stable sites of EMT cortex tethering

**Fig 1:**
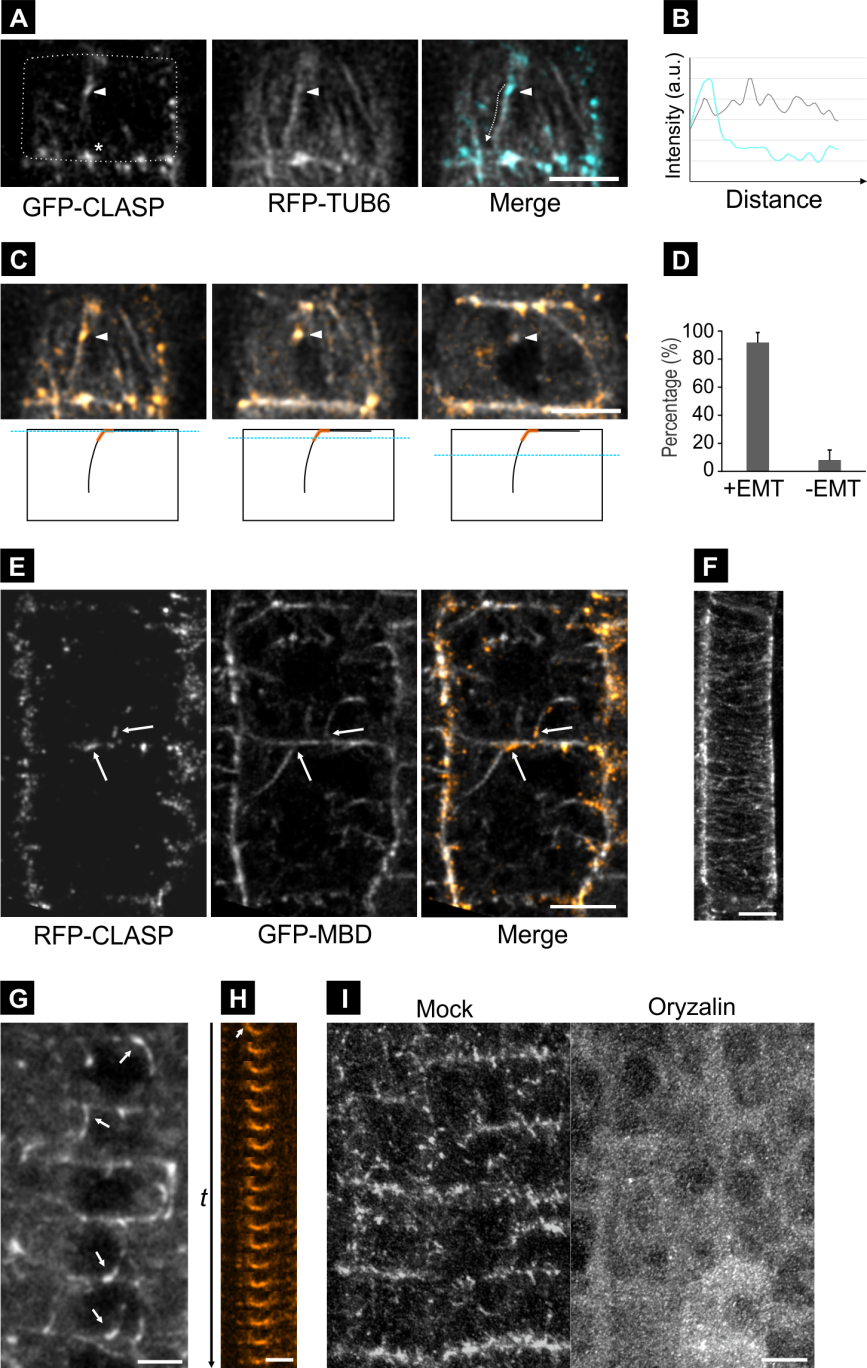
CLASP is enriched at EMT-cortex anchor points. **A.** Surface view of a root tip epidermal cell co-expressing pCLASP::GFP-CLASP (cyan) and pUBQ1::RFP-TUB6 (gray). EMTs often extend into the cortex, and often link to GFP-CLASP at cell edges. Dotted lines indicate cell outline. Cell edge localization is indicated by asterisk. Bar, 5 μm. **B.** Fluorescence Intensity profile plot corresponding to the EMT-cortex anchor point from panel A. Dotted arrow is drawn next to the region for the plot for reference. Grey line = MTs; Cyan line = CLASP. **C.** Confocal sections through the cell in A, starting at the surface and moving in at 0.35 μm increments. Arrowhead traces an EMT into the cell interior. The position of each section is indicated by dot blue line in the illustration below. The square box, black line, and orange line illustrate root cell, EMT, and CLASP, respectively. **D.** Quantification of GFP-CLASP/RFP-TUB6 association. (n = 5 roots, 25 cells, and 316 CLASP spots). p < 0.01, Student’s t test. **E.** Confocal sections through the cell mid-planes of root tip epidermal cells co-expressing *CLASP*::*TagRFP-CLASP* and *UBQ1*::*GFP-MBD*, showing enrichment of RFP-CLASP (orange) at sites corresponding to EMT-cortex attachment points (arrows). MTs are visualized by GFP-MBD (grey). Bar, 5 μm. **F.** GFP-CLASP localization in an elongating root epidermal cell. GFP-CLASP decorates CMTs along their lengths. Cell edge and EMT-anchor enrichment is absent. Bar, 5 μm. **G.** Confocal sections through cell mid-planes of division stage cells with strong GFP-CLASP accumulation along EMT bundles (arrows). Bar, 5 μm. **H.** Time series montage showing GFP-CLASP enrichment at sites of stable EMT-cortex attachment (arrow). Intervals between frames is 32 sec, and total time is 16 minutes. Bar, 2.5 μm. **I.** Root tip epidermal cells expressing GFP-CLASP treated with oryzalin (50 μm, 45min) or mock (45min, 0.5% DMSO). Bar, 5 μm.

In root meristematic cells, in addition to CLASP’s MT localization and cell edge enrichment, we observed numerous CLASP-labelled structures of varied shape and size associated with CMTs at non-edge regions of the cell cortex (Fig 1A). Co-labelling between GFP-CLASP and MTs using RFP-TUB6 revealed that these spots coincide with sites where endoplasmic MTs attach to the cell cortex and incorporate into the CMT array (Fig 1A, 1B and 1C). Quantification showed that 92% ± 7% of CLASP cortical spots corresponded to EMT-cortex attachment sites (Fig 1D; n = 25 cells, 316 cortical CLASP spots).

The same pattern was observed using a native promoter-driven RFP-CLASP fusion and the MT reporter GFP-MBD, which, owing to its lower cytosolic fluorescence compared to RFP-TUB6, allows clearer images from deeper into the cell, where EMTs can be seen attached to sites on the transverse and radial walls (Fig 1E, arrows).

The EMT-cortex labelling pattern of CLASP is distinct from that seen in mature vacuolated cells of the root epidermis, where it decorates CMTs along their length, and is also not enriched at cell edges (Fig 1F). In general, the higher the levels of GFP-CLASP, the more pronounced the EMT labelling was. In cases where EMTs exist as bundles, higher levels of CLASP were present at cortex attachment sites, where it formed hook-like structures, with CLASP signal often extended partially into the cytoplasm along unattached regions of EMTs (Fig 1G). In contrast to the rapid dynamics of CMT ends, EMT tethering sites were relatively long-lived and exhibited little lateral movement over time (Fig 1H).

In order to determine if MTs are required for CLASP localization at EMT-cortex attachment sites, we treated plants expressing GFP-CLASP with the MT-depolymerizing drug oryzalin. This resulted in rapid loss (<30 minutes) of GFP-CLASP from EMT-cortex sites, as well as cell edges (Fig 1I), indicating that MTs are required for recruitment of CLASP to these areas.

### CLASP is required for normal EMT organization in root meristematic cells

**Fig 2:**
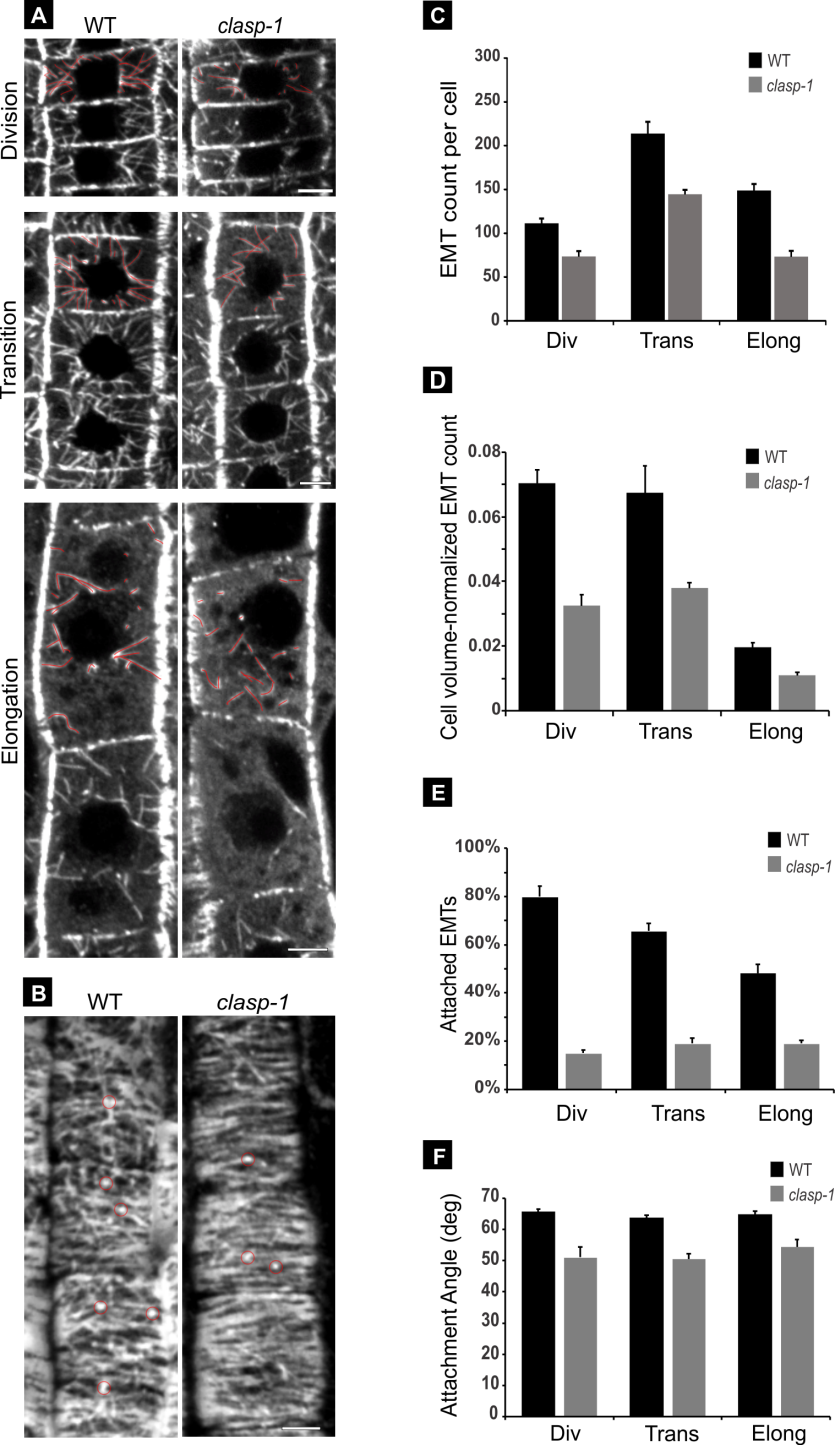
EMT phenotypes in *clasp-1* plants. **A.** Anti-tubulin immunofluorescence images of root tip epidermal cells of wild type (left) and *clasp-1* (right). EMT abnormalities are seen in all three developmental stages. Cellular mid-planes are shown for each stage, using average Z-projections. Division stage shows average projections of 4 Z-slices, corresponding to 2 μm. Transition and elongation stages show average projections of 6 Z-slices corresponding to 3 μm. Bars, 5 μm. **B.** Anti-tubulin immunofluorescence images at the outer epidermal surface of root tip epidermal cells of wild type (left) and *clasp-1* (right). Images are average projections of 4 Z-slices, corresponding to 2 μm. Bar, 5 μm. **C.** Total EMT numbers per cell in wild-type and *clasp-1* root tip epidermal cells. EMT numbers are reduced in all three developmental stages in *clasp-1* compared to wild type. n = 24 cells for each genotype; 800 ~ 1700 total EMTs wild type, 600 ~ 1000 EMTs in *clasp-1*; 3 roots in wild type and 5 roots in *clasp-1*. **D.** EMT numbers normalized for cell volumes (EMT number/cubic μm) in each developmental zone of wild type and *clasp-1*. Taking into account the larger cell volumes of *clasp-1*, the EMT reductions are more severe. **E.** Percentage of attached EMTs in *clasp-1* compared to wild type in each developmental zone. n = 24 cells for each genotype; n = ~600 EMTs *clasp-1* and ~1000 EMTs WT for each cell stage. **F.** The EMT attachment angles in *clasp-1* are smaller than in wild type in each zone of the root tip. n = 12 cells each genotype, n = 60~100 EMTs *clasp-1* and 300~400 EMTs WT for each cell stage.

To determine the function of CLASP at EMT-cortex anchor points, we compared EMT organization in *clasp-1* and wild-type roots using anti-tubulin immunofluorescence. As shown in Fig 2, *clasp-1* showed strong defects in EMT organization throughout all developmental regions of the root tip (see also S1 Movie). Specifically, there were marked reductions in both the number of EMTs (Table 1), as well as the proportion of EMTs that were attached to the cell cortex (Table 2). Fig 2A shows this via cellular mid-plane images of epidermal cells in WT and *clasp-1*, and Fig 2B shows the outer epidermal surface, where EMT attachment sites appear as spots within the cortical array.

**Table 1:** EMT organization in WT and *clasp-1*.

Genotype	Cell stage	EMT number ^a^	EMT number/Cell volume ^b^	EMT length (μm) ^c^
WT	Division	111.3 ± 5.5	0.07 ± 0.004	3.77 ± 0.08
	Transition	213.9 ± 13.3	0.07 ± 0.008	4.13 ± 0.06
	Elongation	148 ± 7.4	0.02 ± 0.001	3.88 ± 0.07
*clasp-1*	Division	73.5 ± 6.1	0.03 ± 0.004	3.75 ± 0.11
	Transition	144.5 ± 5.0	0.04 ± 0.002	3.82 ± 0.07
	Elongation	73.4 ± 6.6	0.01 ± 0.001	3.20 ± 0.09

Data are means ± SE

^a & b^ n = 24 for each genotype

^c^ n = 24 cells for each genotype, n = 600~1000 EMTs *clasp-1* and 800~1700 EMTs WT for each cell stage

p < 0.01, Student’s t test

**Table 2:** EMT-cortex attachment in WT and *clasp-1*.

Genotype	Cell stage	Attached EMTs (%) ^a^	EMT-cortex anchoring angle (Deg.) ^b^
WT	Division	80 ± 4	65.6 ± 0.9
	Transition	66 ± 3	63.7 ± 0.9
	Elongation	48 ± 4	64.7 ± 1.1
*clasp-1*	Division	15 ± 2	51.0 ± 3.3
	Transition	19 ± 2	50.6 ± 1.7
	Elongation	19 ± 1	54.4 ± 2.2

Data are means ± SE

^a^ n = 24 cells for each genotype; n = ~600 EMTs *clasp-1* and ~1000 EMTs WT for each cell stage

^b^ n = 12 cells each genotype, n = 60~100 EMTs *clasp-1* and 300~400 EMTs WT for each cell stage

p < 0.01, Student’s t test

To accurately assess and quantify these EMT defects, a number of caveats must be taken into account in order to accurately define the growth status of a given cell. First, there is a normal gradual depletion in EMTs as cells mature [7]. Second, hair-forming cells (trichoblasts) and non-hair forming cells (atrichoblasts) form distinct files within the root epidermis, and do not initiate elongation and vacuolation synchronously [42–44]. Third, *clasp-1* mutants have smaller meristems due to fewer division zone cells, and shorter cell lengths at maturity [20,30]. All of these factors will influence measurements of EMT organization. To bypass any bias in defining distinct developmental “zones” along the root tip, classifications were instead made on a cell-by-cell basis in order to accurately assess their individual growth states. Cells were thus classified into division, transition, and elongation stages. Division stage cells have not undergone any apparent elongation, transition stage cells have just begun elongation and are roughly square in medial sections, and elongation stage cells are distinguished by their large length: width aspect ratio, the appearance of a large vacuole, and highly transverse CMT arrays.

For quantification, we measured total EMT numbers per cell and EMT lengths using the 3D tracing software 3Dmod [45]. Surprisingly, the lengths of EMTs showed no apparent differences (S1 Fig and Table 1). However, EMT numbers in *clasp-1* showed an approximate 30% reduction in division and transition stage cells, and a ~50% reduction in elongation stage cells compared to wild type (Fig 2C and Table 1). Since the volume of *clasp-1* cells in each cell stage often differs from that of wild-type cells, we performed additional calculations of EMT numbers within a cell as a function of that cell’s volume. This revealed that pre-normalization measurements were moderately underestimated in division and transitioning cells (~50% reduction compared to ~30%), but similar in elongating cells (~50% reduction in *clasp-1*) (Fig 2D and Table 1).

In addition to the reduced EMT abundance, we found a large reduction in the percentage of EMTs that were attached to the cell cortex in *clasp-1* (Fig 2E and Table 2). In wild-type, the abundance of attached EMTs gradually drops as cells mature. However, *clasp-1* had roughly similar numbers for the three stages. Using 3Dmod, we directly quantified the percentage of EMTs attached to the cell cortex (Summarized in Table 2). In division stage cells, 80% of EMTs were attached in wild type, compared to 15% in *clasp-1*. In transition stage cells, 65% of EMTs were attached in wild type, compared with 19% of attached EMTs in *clasp-1*. In elongation stage cells, 48% of EMTs were attached in wild type, compared with 19% in *clasp-1* (Fig 2E and Table 2).

Interestingly, in *clasp-1* cells, the small percentage of EMTs that were attached to the cell cortex tended to do so at shallower angles than wild type. For quantification of EMT attachment angles, we defined a straight-on attachment between EMT and cortex as 90°. For all cell stages, the attachment angle of EMTs in *clasp-1* was roughly 50° compared to 65° in wild type (Fig 2F and Table 2).

### CLASP promotes attachment and stable tethering of EMTs to the cell cortex

**Fig 3:**
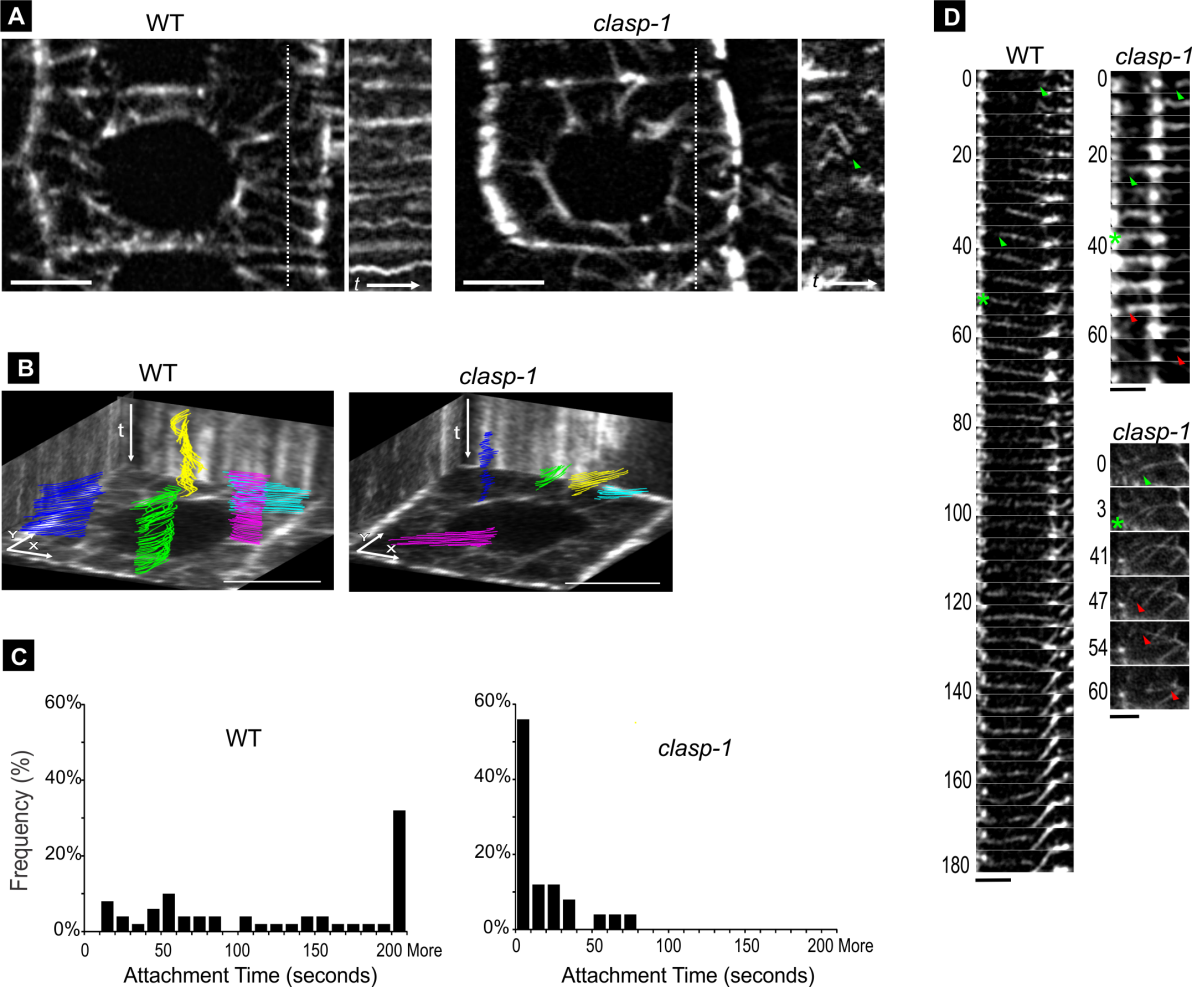
Dynamics of EMT-cortex attachment in *clasp-1*. **A.** Single time point image in the mid-plane of transition zone cell in both wild type (left) and *clasp-1* (right) expressing *UBQ1*::*GFP-MBD*. The kymograph on the right side of each image correspond to the white dotted line. Arrowhead indicates EMT with lateral mobility in *clasp-1*. Time course t = 200 second (4 second intervals). Bars, 5 μm. **B.** Life histories of individual EMTs from a transition zone cell over the course of 200 second (4 second intervals). Different coloured traces correspond to individual. Images are tilted 3D projections. The rear and side walls are kymographs for spatial and temporal reference. Bar, 5 μm. **C.** Histogram showing EMT-cortex attachment times for wild type and *clasp-1*. Since most EMTs remained attached beyond the duration of the observation in wild type, these are grouped as ‘more’. n = 5 roots each genotype, 5 cells per root, 100 EMTs. **D.** Time series images showing establishment of stable EMT-cortex attachment in wild type and failed attachments in *clasp-1*. Green arrowheads indicate growing ends; red arrowheads indicate shrinking ends; asterisks indicate onset of EMT encounter with cortex (on left). Two examples of clasp-1 are shown. Top panel is brief encounter without MT bending, and bottom panel is brief encounter with bending. The time series is 180s in wild type and 70s in *clasp-1* top panel, and 60s in *clasp-1* bottom panel. Bars, 3μm.

To determine the basis for the EMT defects in *clasp-1*, we performed live-cell imaging on root tip division and transition zone cells of *clasp-1* and wild type stably expressing *UBQ1*::*GFP-MBD*. In wild type, EMTs typically grow outward through the cytoplasm until reaching the cell cortex, where they attach, become stably tethered, and show very little lateral movements (illustrated by continuous straight lines in the kymograph in Fig 3A and by the “3D-kymograph” in Fig 3B). The attachment time of EMTs in wild type is quite stable, usually exceeding our observation periods of 200–250 seconds (2.5 second acquisition intervals; Fig 3C). In *clasp-1* however, upon encounter with the cortex, EMTs failed to form stable attachments, instead undergoing catastrophe and depolymerization (Fig 3A–3D; S2 Movie). Depolymerization and detachment were often preceded by a brief pause, and in many cases, bending and swinging away from the site without depolymerization (Fig 3D). Untethered EMTs in *clasp-1* exhibited significant lateral mobility (i.e. waving around) within the cytoplasm, and showing continuous “searching” behaviour as plus ends switched between growth and shortening upon cortex encounter. Viewing S2 Movie will be useful for the reader, as these behaviours are difficult to illustrate with still images.

### *clasp-1* mutants have dominantly globular-shaped vacuoles and enhanced cytoplasmic dynamics

**Fig 4:**
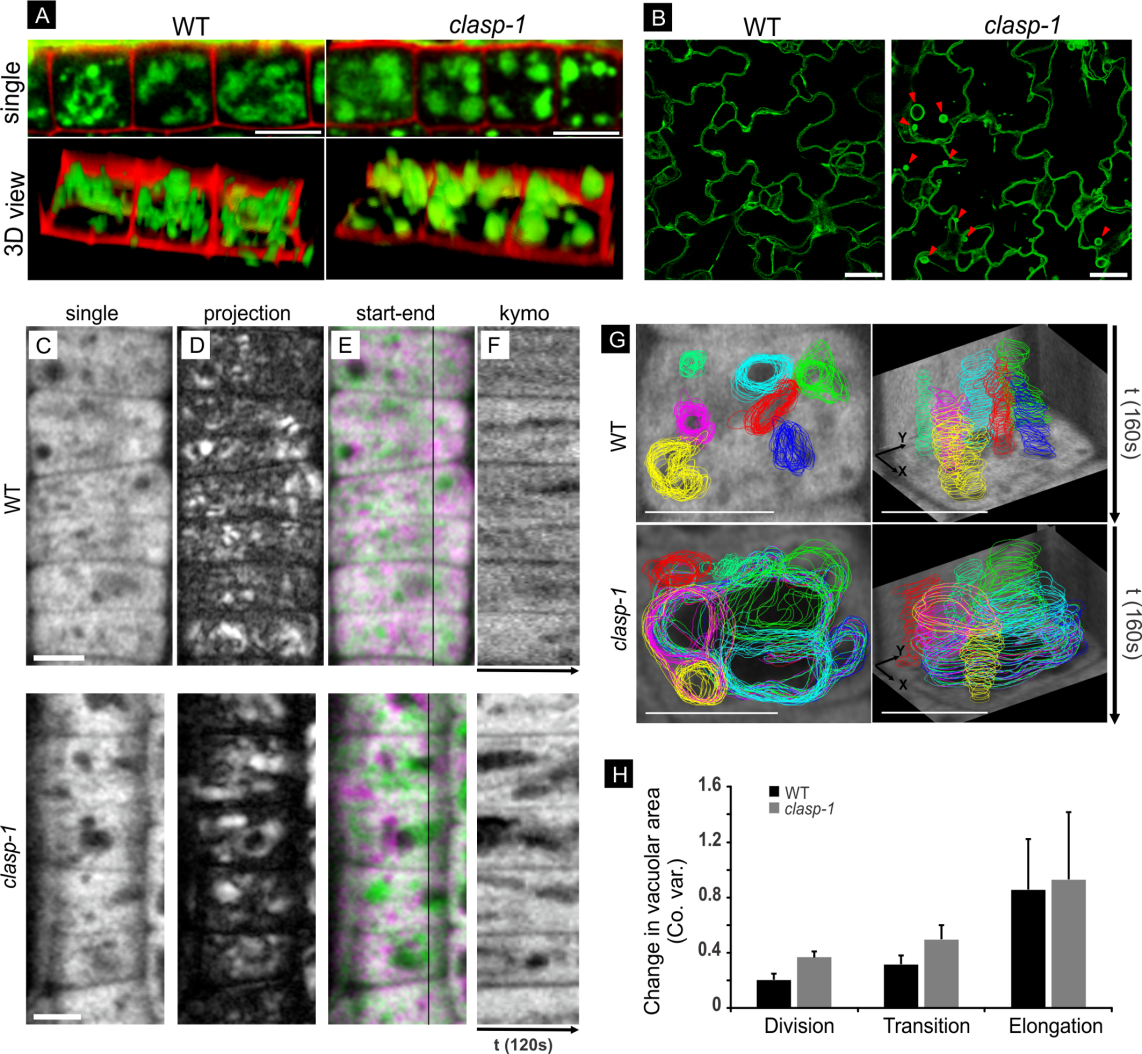
Globular vacuole appearance and cytoplasmic instability in *clasp-1* mutant. **A.** Vacuole morphology of transition stage cells were visualized by BCECF (100μM) and presented in single view (top) and 3D view (bottom) in both wild type and *clasp-1*. Red is propidium iodide. Bars, 10μm. **B.** Tonoplast morphology in expanding cotyledon cells of wild-type and *clasp-1* plants expressing *35S*::*GFP-ɣTIP*. Arrowheads indicate spherical dilations of vacuolar membranes in *clasp-1*. Bars, 10μm. **C.** Single time-point image of epidermal root division zone cells from wild-type and *clasp-1* plants expressing *UBQ1*::*eGFP*. Bar, 5 μm. **D.** Time projection (standard deviation method) of cells in A showing the enhanced vacuole/cytoplasm movement in *clasp-1*. White areas indicate regions that underwent positional/morphological change over the course of observation. Images correspond to 120 second time-lapses, 4 second intervals. **E.** Color merge of two time points from same time series. Green is start and magenta is t = 120 seconds later. White areas indicate no movement between time points. **F.** Kymographs corresponding to the black line in the two-colour merged images in C. The enhanced cytoplasmic movements of *clasp-1* appear as large dark areas of variable size, position, and duration. Total time is 120 seconds. **G.** Life histories of several vacuoles from transitioning cells of wild type and *clasp-1*. Different coloured traces correspond to individual vacuoles. Left image shows all time-points projected onto a single image frame for reference. Right images are tilted 3D projections. The rear and side walls are kymographs for spatial and temporal reference. Total time for each series is 160 second (4 second intervals). Bar, 5 μm. **H.** Variations in vacuolar area over time in wild-type and *clasp-1* root epidermal cells. Time series were acquired at 4 second intervals for 80 seconds, and vacuolar coefficients of variation (co. var.) were calculated for each vacuole. The bars show the means ± SE of 30 vacuoles for each genotype in each cell stage.

In meristematic cells, cytoplasmic streaming is normally minimal, and vacuoles show a tubular organization. As cells prepare for elongation, these tubular vacuoles gradually enlarge and take on a globular appearance [46]. Given CLASP’s involvement in endomembrane trafficking [27] and its role in blocking premature cell elongation in meristematic cells [20,30,36], we hypothesized that the detached and waving EMTs in *clasp-1* lead to defects in cytoplasmic stability and premature vacuolation. To visualize vacuole structure, we stained roots with 100μM BCECF (vacuolar lumen) and propidium iodide (cell wall). Fig 4A shows that *clasp-1* plants indeed show a reduction in tubular vacuoles and a corresponding enhancement of the globular type. To directly visualize vacuolar membranes (VMs), we used wild-type and *clasp-1* plants expressing the tonoplast marker ɣTIP-GFP [37,47]. Because this reporter showed too much non-specific membrane labelling in root tips, we used expanding cells from young cotyledons, where its localization is distinct and specific to VMs (Fig 4B). In both wild-type and *clasp-1* cells, ɣTIP-GFP labelled the tonoplast at the cell periphery and in transvacuolar stands. In *clasp-1* however, we also observed large spherical dilations of VM both within transvacuolar strands and at the cell periphery (Fig 4B, arrowheads).

To visualize cytoplasmic dynamics, we used wild-type and *clasp-1* plants expressing free GFP under control of the *UBQ1* promoter. The exclusion of GFP from vacuoles provided confirmation of the enlarged globular vacuoles in *clasp-1*, as observed with BCECF and ɣTIP-GFP. Compared to the dense and relatively stable cytoplasm in wild-type meristematic cells, the cytoplasm in *clasp-1* cells was more mobile and unstable (Fig 4C–4F). This enhanced dynamicity is illustrated in Fig 4 and S3 Movie, which show plants expressing *UBQ1*::*eGFP* to visualize the cytoplasm and vacuoles. Fig 4 shows images from time series processed as: (C) single time point for reference; (D) time projections (standard deviation method), (E) two-color merges of two time points; and (F) kymographs. Standard deviation projections turned out to be a very good method for assessing cytoplasmic movement, as areas showing change over time appear white, while stable regions appear black. As seen in Fig 4D, *clasp-1* has brighter and larger white areas compared to wild-type cells of the same stage and size. Similarly, the two-color merges of *clasp-1* show enhanced magenta or green areas, indicating movement between the two time points. In kymographic analyses, the enhanced cytoplasmic dynamicity in *clasp-1* appears as sloping variations in fluorescence intensity, with greater variations in size and length of dark regions, as compared to the relatively stable and straight lines in wild type (Fig 4F).

For quantitative analysis of cytoplasmic dynamics, we tracked changes in vacuolar morphology over time in GFP-expressing wild-type and *clasp-1* plants. Fig 4G shows projections of 160-second time lapses with visibly distinct regions of vacuoles color-coded and traced over time (see also S3 Movie). Using 3Dmod for tracing also allows time projections to be tilted for a three-dimensional perspective (i.e. “3D kymographs”; right panels). The numerical data from these traces was then used for quantitative analysis of cytoplasmic movement. To this end, we measured changes in individual vacuolar areas over time as a proxy for cytoplasmic movement. Temporal variation in vacuolar area is expressed as standard deviation of the mean (coefficients of variation) vacuole area to normalize for the large range in areas between individual vacuoles (Fig 4H; see legend for details). As shown in Fig 4H and Table 3, temporal variation in vacuole areas in *clasp-1* was roughly 1.9x higher than wild type in division stage cells, 1.6x higher in transition stage cells. In contrast, elongation stage cells showed no significant difference between wild type and *clasp-1*.

**Table 3:** Coefficient of variation of vacuole area.

Cell zone	WT ^a^	*clasp-1* ^b^	0.5% DMSO^c^	50 μm Oryzalin ^d^
Division	0.20 ± 0.04	0.36 ± 0.04	0.30 ± 0.03	0.70 ± 0.16
Transition	0.32 ± 0.06	0.50 ± 0.10	0.38 ± 0.06	0.72 ± 0.08
Expand	0.86 ± 0.36	0.93 ± 0.48	1.28 ± 0.30	1.00 ± 0.22

Data are means ± SE

^a & b^ n = 30 vacuoles for each genotype

^c & d^ n = 30 vacuoles for each genotype

p < 0.01, Student’s t test

### MT depolymerisation causes the appearance of globular vacuoles and increased cytoplasmic dynamics

**Fig 5:**
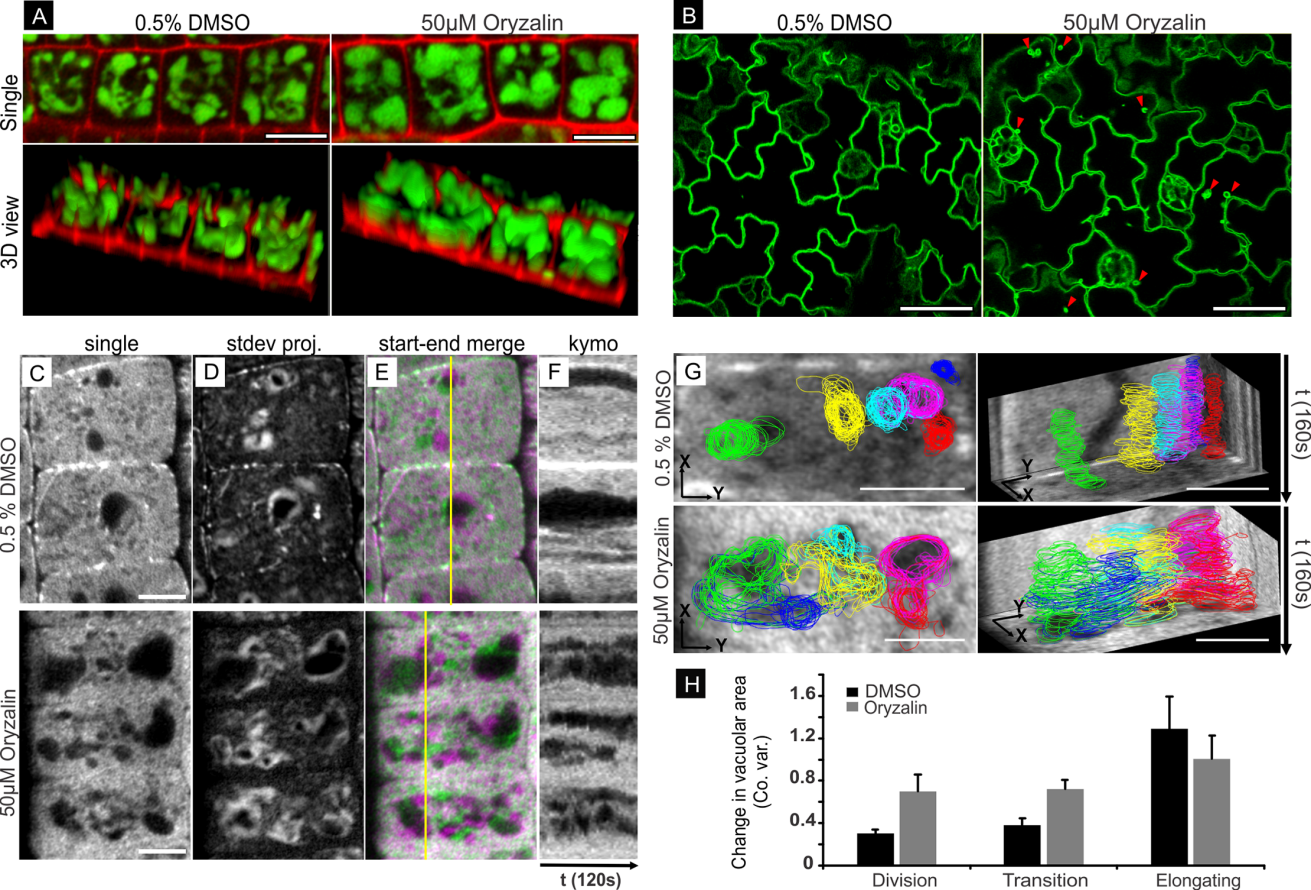
Globular vacuole appearance and cytoplasmic instability in wild-type plants treated with oryzalin. **A.** Single view (top) and 3D view (bottom) of vacuoles in wild-type transition stage cells. Vacuoles were visualized following staining with 10μm BCECF staining (10 μm) of mock (0.5% DMSO) and 50μm oryzalin-treated roots. Red is propidium iodide to visualize cell walls. Bars, 10μm. **B.** Cotyledon epidermal cells from wild-type plants expressing 35S::GFP-ɣTIP, treated for 30min with 50μm oryzalin (right), or mock 0.5% DMSO (left). Bars, 10μm. **C.** Single time-point images of root epidermal division zone cells from wild-type plants expressing *UBQ*::*GFP-TUB6* treated with mock 0.5% DMSO or 50μm Oryzalin. Images were taken after 45 minutes of treatment. **D.** Time projection (standard deviation method) of cells in A showing the enhanced vacuole/cytoplasm movement in oryzalin-treated plants. White areas indicate regions that underwent positional/morphological change over the course of observation. Images correspond to 120 second time-lapses, 4 second intervals. **E.** Color merge of two time points from same time series. Green is t = 1 and magenta is t = 120. White areas indicate no movement between time points. **F.** Kymographs corresponding to yellow lines in the two-colour merged images. Enhanced dynamicity of vacuoles mirrors that observed in *clasp-1* cells. Total time is120 seconds. Bars, 10μm. **G.** Life histories of individual vacuoles from a transition stage cell over the course of 160 seconds (4 second intervals). Different coloured traces correspond to individual vacuoles. Left image shows all time-points projected onto a single image frame for reference. Right images are tilted 3D projections. The rear and side walls are kymographs for spatial and temporal reference. Bars, 10μm. **H.** Coefficients of variation (co.var.) for vacuole area in control (0.5% DMSO) and roots treated with 50 μm oryzalin. Time between compared time points 80 second. n = 30 vacuoles for each genotype. Bars indicate Standard Error.

The above data suggest that the loss of stable EMT-cortex tethering in *clasp-1* may lead to the enlarged, globular-shaped vacuoles and enhanced mobility of the cytoplasm. To probe this further, we depolymerized MTs in wild-type plants using oryzalin, and then observed vacuole morphology and cytoplasmic dynamics. We used the same staining (BCECF) and lines (*35S*::*ɣTIP-GFP*) as for the comparison between WT and *clasp-1* for vacuole morphology. In order to minimize any downstream pleiotropic effects caused by MT depolymerization, we restricted our observations to within 1 hour of treatment. We found that EMT disruption using oryzalin treatment induced changes in vacuole morphology from tubular- to globular-shaped in WT meristematic root cells (Fig 5A). This response was rapid, becoming apparent after 30–45 minutes in oryzalin. In WT cotyledons expressing ɣTIP-GFP, we found that spherical dilations of tonoplast membrane started to appear after 30–45 minutes of oryzalin treatment (Fig 5B). These data show that depolymerization of MTs with oryzalin lead to similar effects on vacuolar morphology and cytoplasmic mobility as observed with *clasp-1* mutants. However, unlike the *clasp-1* mutation, drug-induced MT polymerization removes not only EMTs, but also CMTs, so we cannot exclude potential effects of CMT depolymerization on vacuole morphology.

In order to visualize the cytoplasm while simultaneously monitoring MT polymer status, we used GFP-TUB6, which shows relatively high levels of cytoplasmic fluorescence from unpolymerized subunits. This also allowed us to minimize treatment times to avoid potential indirect or downstream effects of MT depolymerization. Depolymerization of MTs with 50μM oryzalin resulted in enlarged, globular vacuoles and enhanced cytoplasmic mobility within both division and transition zone cells relative to mock treatment (Fig 5C–5F). This change was evident after short treatment times (30–60 minutes). In standard deviation projections, oryzalin treatment has brighter and larger white areas compared to control DMSO of the same stage and size cells (Fig 5D). Similarly, the two-colour merges of oryzalin treatment show enhanced magenta or green areas, indicating movement between the two time points compared to DMSO control (Fig 5E). In kymographs, the enhanced cytoplasmic dynamicity in oryzalin treatment appears as sloped or varied size and length dark regions, as compared to the relatively stable and straight lines in DMSO (Fig 5F). Fig 5G shows time-compressed images of vacuolar traces, as in Fig 4G (see also S4 Movie).

Quantification showed higher temporal variation in vacuole area (expressed as coefficient of variation) in oryzalin-treated cells compared to mock treatments (Fig 5H). These results show that drug-induced MT depolymerization leads to a *clasp-1*-like effect on vacuole morphology and cytoplasmic dynamics.

In moss, EMTs have been shown to physically associate with vacuoles and induce morphological changes such as tubular protrusions during their polymerization [48]. To test for a similar relationship between EMTs and vacuoles in *Arabidopsis*, we simultaneously visualized EMTs and vacuoles by staining root tips expressing *UBQ*::*GFP-MBD* with FM4-64 (which follows the endocytic pathway from plasma membrane to tonoplast)(S2 Fig). In short, we saw no evidence of EMT dynamics causing vacuole tubulation or contortion (for example, a growing EMT plus-end associating with and pulling an extended tubular structure), and there was no obvious overall pattern of association between EMTs and vacuoles. Some EMTs showed no contact with vacuoles, while others did appear to have sites of lateral contact with vacuoles at some point(s) along the EMT sidewall (Panels A and B in S2 Fig; S5 Movie). These lateral contact sites did not appear to correlate with vacuolar morphology, and were often small relative to the associated vacuole. On the other hand, lateral contacts were often stable over the course of minutes, and showed coordinated movement over short distances in the lateral dimension (Panels C and D in S2 Fig; S6 Movie), although this apparent relationship may merely be a result of two dense cytoplasmic components responding to the same local actomyosin-based cytoplasmic movement. These results suggest an indirect role for EMTs in influencing vacuole morphology and dynamics.

## Discussion

Our data show that CLASP is required for EMT-cortex tethering, and suggest that this tethering is required to stabilize the cytoplasm from cytoplasmic streaming and vacuolation. To our knowledge, this is the first characterization of a molecule with this localization pattern and function in plants. CLASP’s role in stabilizing radial EMT arrays in plants is remarkably similar to the case in animal cells, where CLASP localizes at the cell cortex to provide local stabilization to the plus-ends of EMTs that radiate outward from the centrosome [34,49]. A key difference is that in plants, when an EMT plus-end encounters the cell cortex, it can bend and enter into the cell cortex, where it remains laterally anchored to the plasma membrane, thereby establishing a continuous CMT-EMT system. In animal cells, EMTs radiate out from the centrosome, but do not curve into the cell cortex to form cortical arrays. Instead MT plus-ends are stabilized near the cell membrane, where CLASPs promote MT pause and rescue [25, 49–51]. Despite a likely role for plant CLASP in directly influencing MT dynamic instability in plants, evidence to date suggests that CLASP primarily influences CMT organization indirectly by modulating MT-plasma membrane association [26,52]. Interestingly, our data also show that in *clasp-1*, the EMTs that do form attachments with the plasma membrane do so at a smaller angle of incidence than normal, indicating that the compressive forces associated with end-on collisions of EMTs with the membrane may cause depolymerization, as in the case with cell edges [36]. Alternatively, these EMTs may represent partially detached regions of CMTs, as observed in *clasp-1* mutant cotyledon cells [26].

Perhaps because of these differences, the other molecules known to form complexes with CLASP during EMT-cortex stabilization in animals appear to be lacking in plants [20]. While it is unknown if this lack of homologs explains the difference in the manner of EMT-cortex association between plants and animals is unknown, one may speculate that additional plant-specific proteins likely complex with CLASP at EMT-cortex attachment sites, where they encourage direct plasma membrane contact and continued growth into the CMT array.

### Functions of EMTs in plants

EMTs in plants are generally accepted to play a role in generating mixed CMT array orientations, which promotes the characteristic isodiametric cell expansion phase in post-cytokinetic cells [7,18,53]. As cells mature, a gradual loss of EMTs correlates with the establishment of transverse CMT arrays and central vacuoles, both of which facilitate rapid cell elongation. Aside from facilitating CMT array repopulation and mixing in post-cytokinetic cells, little is known as to additional roles of EMTs.

With respect to a possible role of EMTs in cytoplasmic stabilization, evidence to date is mostly circumstantial. First, there is an inverse correlation between EMT abundance and cytoplasmic streaming [54]. In meristematic cells with dense EMT arrays, cytoplasmic streaming is typically minimal, instead showing jiggling or saltatory motions. In contrast, rapidly streaming cells typically contain large, dynamic vacuoles with numerous transvacuolar strands. Our data show that EMT-cortex attachment is lost prior to disintegration of EMTs (Fig 2), suggesting that cortical tethering stabilizes EMTs. In support of this, in tip growing cells such as root hairs, EMTs are present, but not well-tethered to the cell cortex, and these cells have very dynamic cytoplasm and large vacuoles [8]. Furthermore, it has been shown that CMTs are generally more resistant to the MT-disrupting drug colchicine than are EMTs, suggesting that attachment to the cell membrane may indeed provide some degree of stabilization to MTs [55]. *Clasp-1* mutants, which have reduced CMT-cortex attachment and destabilized EMTs, are hypersensitive to oryzalin treatment [20,26], although this may also be due to more generalized reductions in MT stability in *clasp-1*.

While streaming is a characteristic of vacuolated cells, it is unclear whether streaming is required for formation of central vacuoles. With respect to actin organization in division/transition stage cells, these cells have mostly fine actin, and lack the large actin cables associated with cytoplasmic streaming in mature cells [56]. Removal of F-actin with Latrunculin B leads to small spherical vacuoles instead of a central vacuole [57]. Conversely, treatment of root meristematic cells with the auxin NAA leads to an increase in fine actin and disrupts the central vacuole, creating smaller and more convoluted vacuoles, although an influence on cytoplasmic movement was not documented [57]. Interestingly, the *botero-1* mutant of KATANIN, which has increased EMT abundance, is hypersensitive to treatment with Latrunculin B [58]. Whether this is due to a direct actin-EMT relationship or to a more generalized susceptibility due to pleiotropic MT-based defects is not known.

With respect to a relationship between EMTs and vacuoles, studies on the moss *Physcomitrella patens* have shown a close association between EMTs and vacuolar membranes [48]. Specifically, growing EMT plus-ends attached to vacuole membranes and physically “pulled” on the membrane to produce vacuole protrusions. Furthermore, the study showed that oryzalin treatment caused vacuoles to lose their normal tubular shape, becoming enlarged and more spherical, while depolymerization of actin with Latrunculin B had no effect on vacuolar shape or movement. This contrasts to higher plants, where the actin cytoskeleton appears to have taken the dominant role in guiding vacuole shape and dynamics, as well as cytoplasmic and organelle trafficking in general [57, 59–62]. Our data are in agreement with this. While we cannot rule out a direct physical interaction, it is likely that the observed lateral contacts between EMTs and vacuoles may be coincidental as a result of the high densities of EMTs and vacuoles. Because EMTs form stable (both spatially and temporally) connections between the nuclear envelope and the cell cortex, their influence on vacuole morphology may instead be indirect, such as stabilization of the cytoplasm to buffer against cytoplasmic streaming, or by acting as barriers that vacuoles must bend around or remain small to fit in the available open spaces. During entry into expansion phase, the open spaces resulting from loss of EMTs may then allow vacuoles to enlarge.

### A role for EMTs in meristem maintenance?

*clasp-1* mutants have smaller root and shoot apical meristems [20,30,31], a phenotype that is rather unique among MAP mutants, which typically have organ twisting, swollen root tips, and more severe expansion defects [63,64]. This meristem reduction may be explained by the observation that *clasp-1* CMT arrays do not show the typical disorganization associated with many MAP mutants. Instead, *clasp-1* mutants have hyper-parallel CMT arrays in both mature and meristematic cells due to the failure of CMTs to cross over sharp cell edges [36,65]. This is believed to result in premature cell elongation and exit from the meristem. Our current data indicate that the inability to stably tether EMTs to the cortex in *clasp-1* may also contribute to this lack of CMT array mixing, but may also result in destabilized cytoplasm and premature vacuolation, which would also favour cell elongation and exit from the meristem. A third potential influence on meristem size in *clasp-1* mutants is the defects in auxin polar transport and the failure to establish normal auxin gradients in the root tip due to reduced PIN2 recycling [27].

## Supporting information

S1 Fig EMT lengths in wild type and *clasp-1*. Histogram showing EMT length of each cell stage for wild type and *clasp-1*. n = 24 cells for each genotype, n = 600–1000 EMTs *clasp-1* and 800–1700 EMTs WT for each cell stage.
(TIF)

S2 Fig Simultaneous visualization of EMTs (GFP-MBD) and vacuoles (FM4-64). **A.** Serial sections through a Z-series of division stage cells in wild-type root tip expressing *UBQ*::*GFP-MBD* (yellow) stained with 5μM FM4-64 (blue) for 2hr. Each section is a maximum sub-projection of five z-slices tilted for visibility. EMT-vacuole lateral contacts indicated by arrows, and free EMT regions by arrowheads. Bar, 5 μm.
**B.** 3D views of division stage cells from a wild-type root tip expressing expressing *UBQ*::*GFP-MBD* stained with 5μM FM4-64 for 2hr. Corresponds to S5 Movie. Bar, 5 μm.
**C.** Time series montage showing stable lateral association and coordinated movement of EMTs and vacuoles. Intervals between frames is 30 sec and total time is 150 seconds. The dotted circle illustrates vacuole membrane, and the arrow indicates the EMT bundle, which is oriented parallel to the light path. Bar, 5 μm.
**D.** Single time-point image and corresponding kymographs showing stable lateral association between EMTs (cyan), and vacuoles (red). Total time is 3 minutes (time interval = 6s). Bar, 5 μm.
(TIF)

S1 Movie MT organization in WT and *clasp-1* roots. Shown are confocal z-stacks of WT and *clasp-1* root tips immuno-stained for tubulin. EMT traces and 3D projections were made using 3D Mod software.
(AVI)

S2 Movie EMT dynamics in WT and *clasp-1* root division/transition stage cells. MTs are visualized using GFP-MBD. Time series is 200s. Time interval = 4s.
(AVI)

S3 Movie Cytoplasmic dynamics in WT and *clasp-1* root division stage cells. Cytoplasm is visualized here using free GFP. Large dark areas represent vacuoles. Time series is 80s. Time interval = 4s.
(AVI)

S4 Movie Cytoplasmic dynamics in root tips of WT plants treated with oryzalin. Cytoplasmic dynamics in 50 μm Oryzalin treatment and control 0.5% DMSO roots expressing GFP-TUB6 to visualize MTs, cytoplasm and vacuoles. Time series is 120s. Time interval = 4s.
(AVI)

S5 Movie 3D reconstruction of GFP-MBD and FM4-64 in root tip. MTs can be seen both with and without vacuole association. MTs are yellow and vacuoles are blue. Movie recorded in imageJ using ClearVolume plugin.
(AVI)

S6 Movie Dynamics of EMTs and vacuoles in root tip. MTs are visualized using GFP-MBD and vacuoles are visualized using 5 μM FM4-64. The square boxes indicate the coordinated movement of EMTs and vacuoles. Time series is 150s. Time interval = 5s, played at 15 frames per second.
(AVI)
