# Supplementary figures and images for: CLASP promotes stable tethering of endoplasmic microtubules to the cell cortex to maintain cytoplasmic stability in Arabidopsis meristematic cells
Source: PLoS One. 2018 Jun 12;13(6):e0198521. doi: 10.1371/journal.pone.0198521 (PMC5997327; doi:10.1371/journal.pone.0198521)

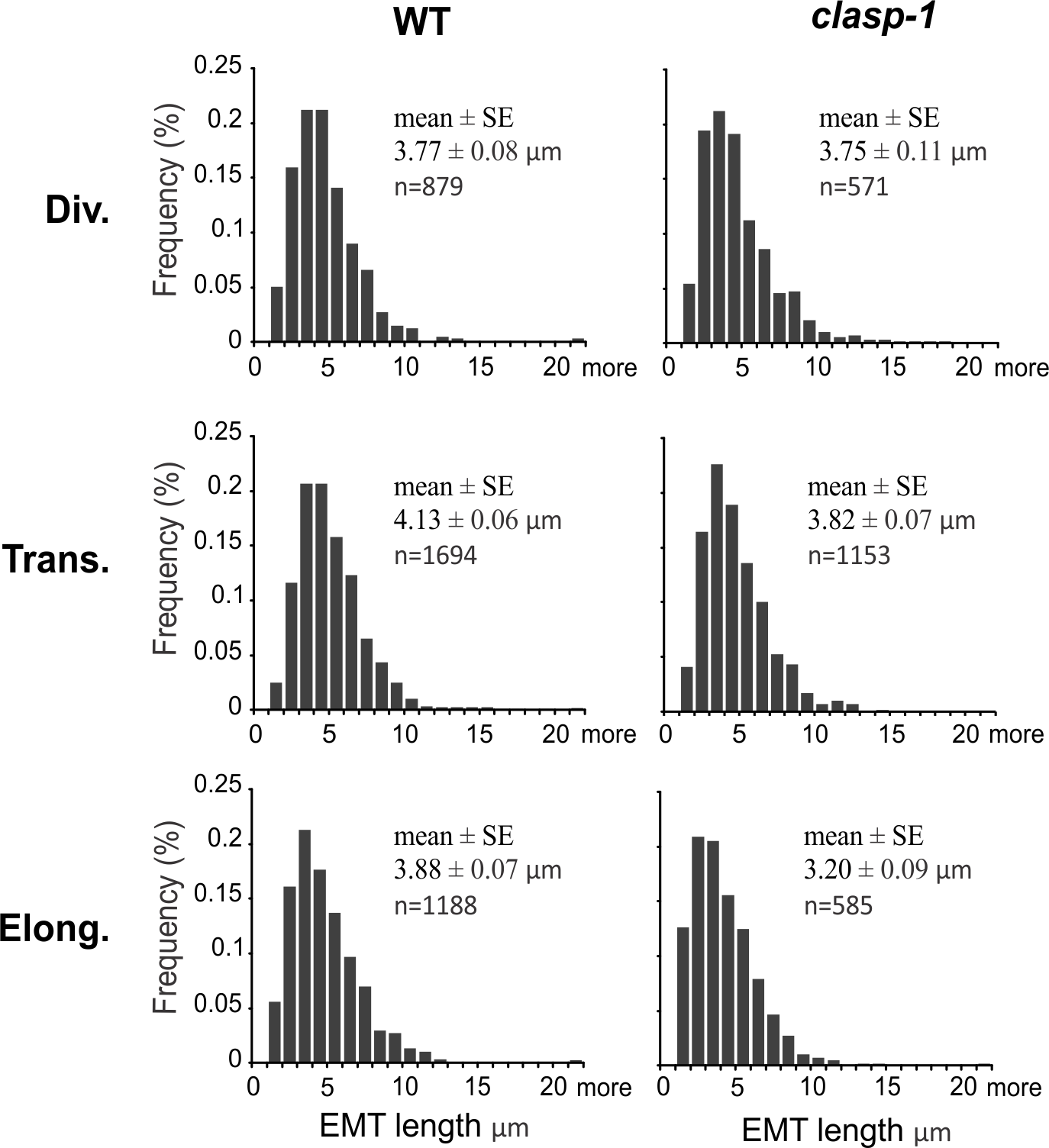

Supplement: S1 Fig — Histogram showing EMT length of each cell stage for wild type and clasp-1. n = 24 cells for each genotype, n = 600–1000 EMTs clasp-1 and 800–1700 EMTs WT for each cell stage. (TIF) [file pone.0198521.s001.tif]

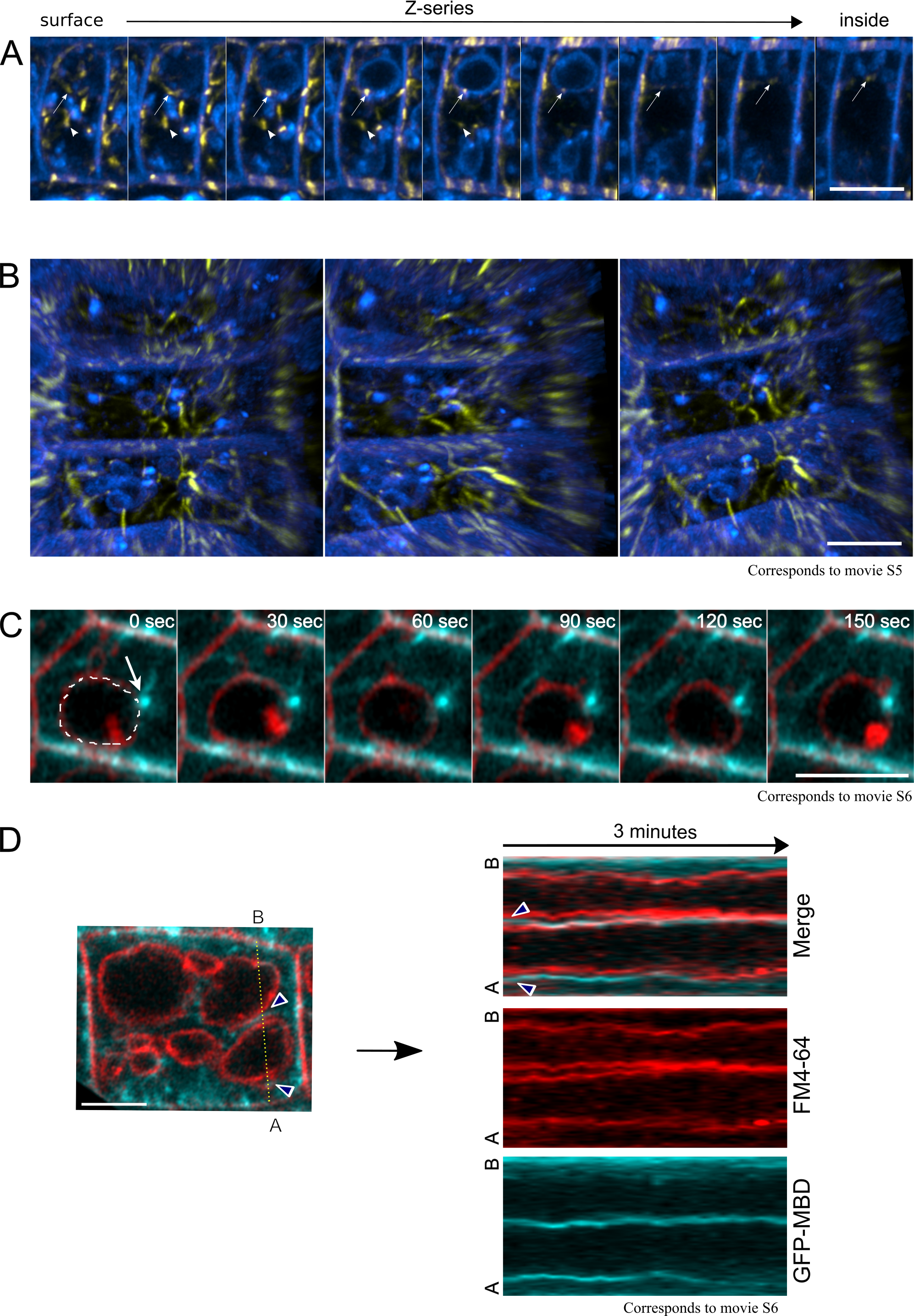

Supplement: S2 Fig — A. Serial sections through a Z-series of division stage cells in wild-type root tip expressing UBQ::GFP-MBD (yellow) stained with 5μM FM4-64 (blue) for 2hr. Each section is a maximum sub-projection of five z-slices tilted for visibility. EMT-vacuole lateral contacts indicated by arrows, and free EMT regions by arrowheads. Bar, 5 μm. B. 3D views of division stage cells from a wild-type root tip expressing expressing UBQ::GFP-MBD stained with 5μM FM4-64 for 2hr. Corresponds to S5 Movie. Bar, 5 μm. C. Time series montage showing stable lateral association and coordinated movement of EMTs and vacuoles. Intervals between frames is 30 sec and total time is 150 seconds. The dotted circle illustrates vacuole membrane, and the arrow indicates the EMT bundle, which is oriented parallel to the light path. Bar, 5 μm. D. Single time-point image and corresponding kymographs showing stable lateral association between EMTs (cyan), and vacuoles (red). Total time is 3 minutes (time interval = 6s). Bar, 5 μm. (TIF) [file pone.0198521.s002.tif]
